# Zero-lag synchronization in cortical motifs

**DOI:** 10.1186/1471-2202-14-S1-P37

**Published:** 2013-07-08

**Authors:** Leonardo L Gollo, Claudio Mirasso, Olaf Sporns, Michael Breakspear

**Affiliations:** 1 IFISC, Instituto de Física Interdisciplinar y Sistemas Complejos (CSIC-UIB), Palma de Mallorca, Spain; 2 Program of Mental Health Research, Queensland Institute of Medical Research, Brisbane, QLD, Australia; 3 Department of Psychological and Brain Sciences, Indiana University, Bloomington, Indiana, USA; 4 School of Psychiatry, University of New South Wales and The Black Dog Institute, Sydney, NSW, Australia; 5 The Royal Brisbane and Woman's Hospital, Brisbane, QLD, Australia

## 

**Figure 1:**
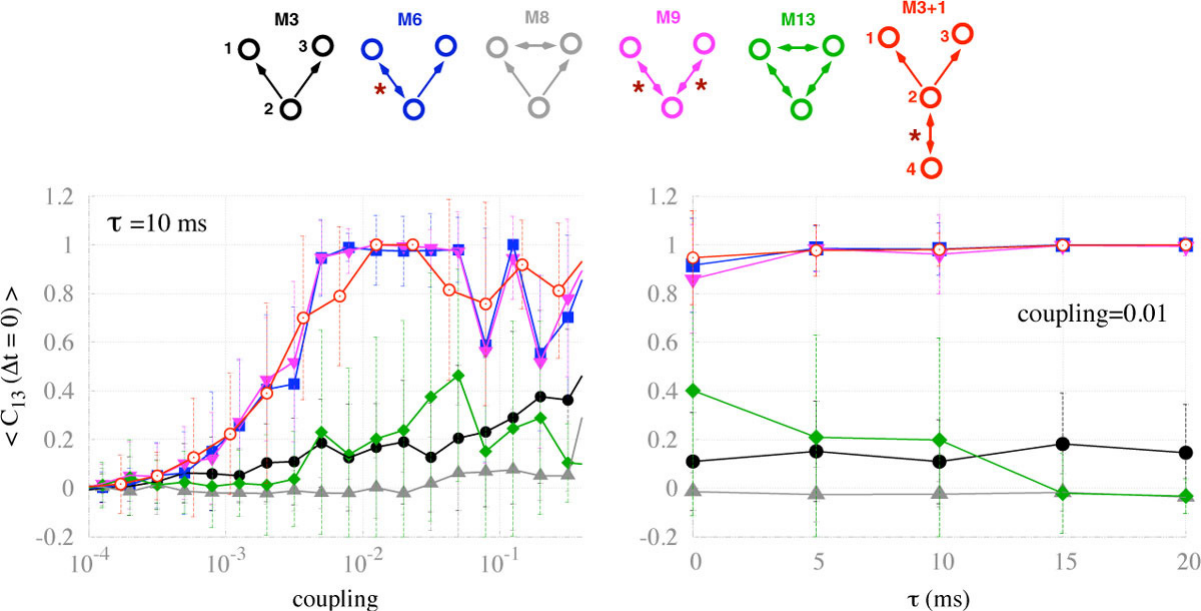
**Motifs and zero-lag cross-correlations between nodes 1 and 3 in mean field neural models for varying coupling strength (left panel) and delay (right panel)**.

Zero-lag synchronization between distant cortical areas has been observed in diverse experimental settings, and between many different regions of the brain [1,2]. Several mechanisms have been proposed to account for such isochronous synchronization in the presence of long conduction delays: Of these, the phenomena of "dynamical relaying" - a mechanism that relies on a specific network motif (M9) - has proven to be the most robust with respect to parameter and system noise [2-4]. Surprisingly, despite a prevailing belief in the community, the common driving motif (M3) is an unreliable means of establishing zero-lag synchrony. Although dynamical relaying has been validated in empirical and computational studies [4], the deeper dynamical mechanisms and comparison to dynamics on other motifs is lacking (see Figure 1). Given the presence of different network motifs in cortical systems, such deeper insights are of high priority. By systematically comparing synchronization on a variety of small motifs, we establish that the presence of a single reciprocally connected pair - a "resonance pair" - plays a crucial role in disambiguating those motifs that foster zero-lag synchrony in the presence of conduction delays (such as dynamical relaying, M9) from those that do not (such as the common driving triad, M3). Remarkably, minor structural changes to M3 that incorporate a reciprocal pair (hence M6, M9, M3+1) recovers robust zero-lag synchrony. The findings are observed in computational models of spiking neurons, populations of spiking neurons and mean field neural models, and arise whether the systems are periodic, chaotic, noise-free or driven by stochastic inputs. The influence of the resonant pair is also robust to parameter mismatch and asymmetrical time delays amongst the elements of the motif. The synchronization of the commonly driven nodes is optimal when the driver node is part of a resonant pair, or is under the influence of another resonant pair (since its effect can propagate in the network). We call this manner of facilitating zero-lag synchrony *resonance-induced synchronization *and propose that it may be a critical feature of zero-lag synchrony in the brain.
